# Management and outcomes in women and men weaning from invasive mechanical ventilation: insights from the WEAN SAFE study

**DOI:** 10.1016/j.aicoj.2026.100037

**Published:** 2026-02-20

**Authors:** Reginald Caldecott, Kate Laffey, Omid Khazaei, Yueyun Zhu, Bairbre A. McNicholas, Emanuele Rezoagli, Tài Pham, Leo Heunks, Giacomo Bellani, Laurent Brochard, Andrew J. Simpkin, Martin Dres, Paolo Navalesi, John G. Laffey, on behalf of the WEAN SAFE Investigators

**Affiliations:** a Anaesthesia and Intensive Care Medicine, School of Medicine, University of Galway, Ireland; b Department of Anaesthesia and Intensive Care Medicine, Galway University Hospitals, Ireland; c School of Medicine, University of Galway, Ireland; d School of Mathematical and Statistical Sciences, University of Galway, Ireland; e Department of Emergency and Intensive Care, Fondazione IRCCS San Gerardo dei Tintori, Monza, Italy; f School of Medicine and Surgery, University of Milano-Bicocca, Monza, Italy; g Service de Médecine Intensive-Réanimation, AP-HP, Hôpital de Bicêtre, DMU CORREVE, FHU SEPSIS, Groupe de Recherche CARMAS, Hôpitaux Universitaires Paris-Saclay, Le Kremlin-Bicêtre, France; h Université Paris-Saclay, UVSQ, University Paris-Sud, Inserm U1018, Equipe d’Epidémiologie Respiratoire Intégrative, CESP, 94807, Villejuif, France; i Department of Intensive Care Medicine, Radboud University Medical Centre, Nijmegen, The Netherlands; j School of Medicine and Surgery, University of Trento, Trento, Italy; k Interdepartmental Division of Critical Care Medicine, University of Toronto, Toronto, Canada; l Keenan Research Centre for Biomedical Science, Li Ka Shing Knowledge Institute, St Michael’s Hospital, Unity Health Toronto, Canada; m Service de Médecine Intensive - Réanimation-SRPR, APHP, Hôpital Pitié-Salpêtrière, Sorbonne Université, 75013, Paris, France; n INSERM, UMRS_1158 Neurophysiologie Respiratoire Expérimentale et Clinique, Sorbonne Université, Paris, France; o Department of Medicine, University of Padua, Padua, Italy, Institute of Anesthesia and Intensive Care, Padua University Hospital, Padua, Italy

**Keywords:** Biologic sex, Woman, Man, Ventilator weaning, Ventilator liberation, Invasive mechanical ventilation

## Abstract

**Objective:**

To understand the differences in the weaning process and outcomes in men and women enrolled in the WorldwidE AssessmeNt of Separation of pAtients From ventilatory assistancE (WEAN SAFE) study.

**Methods:**

We analysed patients in the WEAN SAFE cohort who commenced weaning from invasive ventilation, stratified by biological sex. The primary outcome was the effect of sex on delayed weaning and failed weaning from invasive mechanical ventilation. Secondary outcomes included the influence of sex on ventilatory management, ICU/hospital survival and decisions to limit life-sustaining interventions.

**Results:**

Of 4,523 patients who entered the weaning process, 1,754 (38.8%) were women and 2,769 (61.2%) were men. Women were shorter, had higher P/F ratios, and received higher tidal volumes and lower PEEP than men. Women on controlled ventilation received higher driving pressures, while women on assisted ventilation received higher inspiratory pressures than men. Both female sex and shorter stature were independently associated with higher tidal volume ventilation, with shorter females at particular risk. In univariate analyses, women were less likely to successfully wean from invasive ventilation. When adjusted for factors such as height, age, and frailty profile, there was no independent association between sex and weaning success. In patients with more severe respiratory failure (P/F ratios <200), there were no sex differences in ventilatory support, weaning management and outcomes.

**Conclusions:**

Women weaning from ventilation were shorter and had less severe respiratory failure but received less protective lung ventilation and more frequent ventilatory over-assistance. When adjusted for height and age, female sex was not independently associated with failed weaning from invasive ventilation.

**Trial registration:**

ClinicalTrials.gov, NCT03255109.

## Introduction

Prolonged and failed weaning of patients from invasive mechanical ventilation (MV) presents a significant clinical challenge that profoundly impacts outcomes in the critically ill, increasing intensive care unit (ICU) and hospital stay, and worsening mortality risk [[1], [2], [3], [4]]. There is significant evidence that critically ill women may receive suboptimal care [5,6], with lower ICU admission rates [7], reduced likelihood of receiving invasive ventilation or other organ support [5,7], and potentially worse ICU outcomes [6], compared to men [8,9]. In an Austrian study, women had higher illness severity scores at ICU admission, yet received fewer invasive interventions than men, but ultimately achieved similar survival outcomes [9]. In a large German multi-centre cohort study, men were more likely to receive invasive mechanical ventilation and other organ supports than women with comparable illness severity [10]. In a large Swiss multi-centre ICU cohort, women were less likely to be admitted to ICU, and had a significantly increased risk of death per unit increase in simplified acute physiology score compared to men [6]. In contrast, a large single-centre Swedish cohort study observed that women and men had similar ICU lengths of stay and mortality rates, although women had a higher probability of early ICU discharge, potentially reflecting faster recovery [11].

Critically ill women receive suboptimal ventilatory management, which may complicate the weaning process. Specifically, height-dependent ventilation protocols create systemic challenges, as demonstrated in an analysis of the LUNG SAFE cohort, where females with acute respiratory distress syndrome (ARDS) had poorer outcomes, in part mediated by underuse of lung-protective ventilation in shorter individuals, who are disproportionately female [12]. Similar findings showing underuse of lung-protective ventilation in shorter (mainly female) individuals have been reported in sepsis-induced lung injury [13], and in an analysis of ARDS network trials [14]. Thille et al. reported that that clinicians were more likely to delay extubation in women compared with men, even after successful spontaneous breathing trials (SBTs), potentially impacting outcomes [15]. Women may also have additional risk factors for poorer weaning outcomes, due to physiological differences between men and women in respiratory muscle and diaphragmatic strength [16,17], differences in lung mechanics [18], and anatomical differences including reduced lung volumes and reduced airway size [19].

Because of these issues, we wished to determine the impact of sex differences on the management and outcomes of the weaning process in patients receiving invasive ventilation, in this secondary WEAN SAFE analysis. We hypothesised that women would have poorer outcomes compared to men during weaning from invasive mechanical ventilation.

## Methods and materials

### Study design and setting

This is a pre-defined sub-study of the WEAN SAFE study, an international, multicentre, prospective cohort study of patients undergoing invasive ventilation, conducted during 4 consecutive weeks between October 2017 and June 2018 in a convenience sample of 481 Intensive Care Units (ICUs) from 50 countries, across 5 continents. WEAN SAFE enrolled 5,859 patients that required at least 2 days of invasive MV [1]. The study, jointly supported by the European Society of Intensive Care Medicine and the European Respiratory Society, was endorsed by multiple national societies/networks (Appendix 1). National coordinators and site investigators (Appendix 1) were responsible for obtaining ethics committee approval and for ensuring data integrity and validity.

### Participants and data collection

**Fig. 1:**
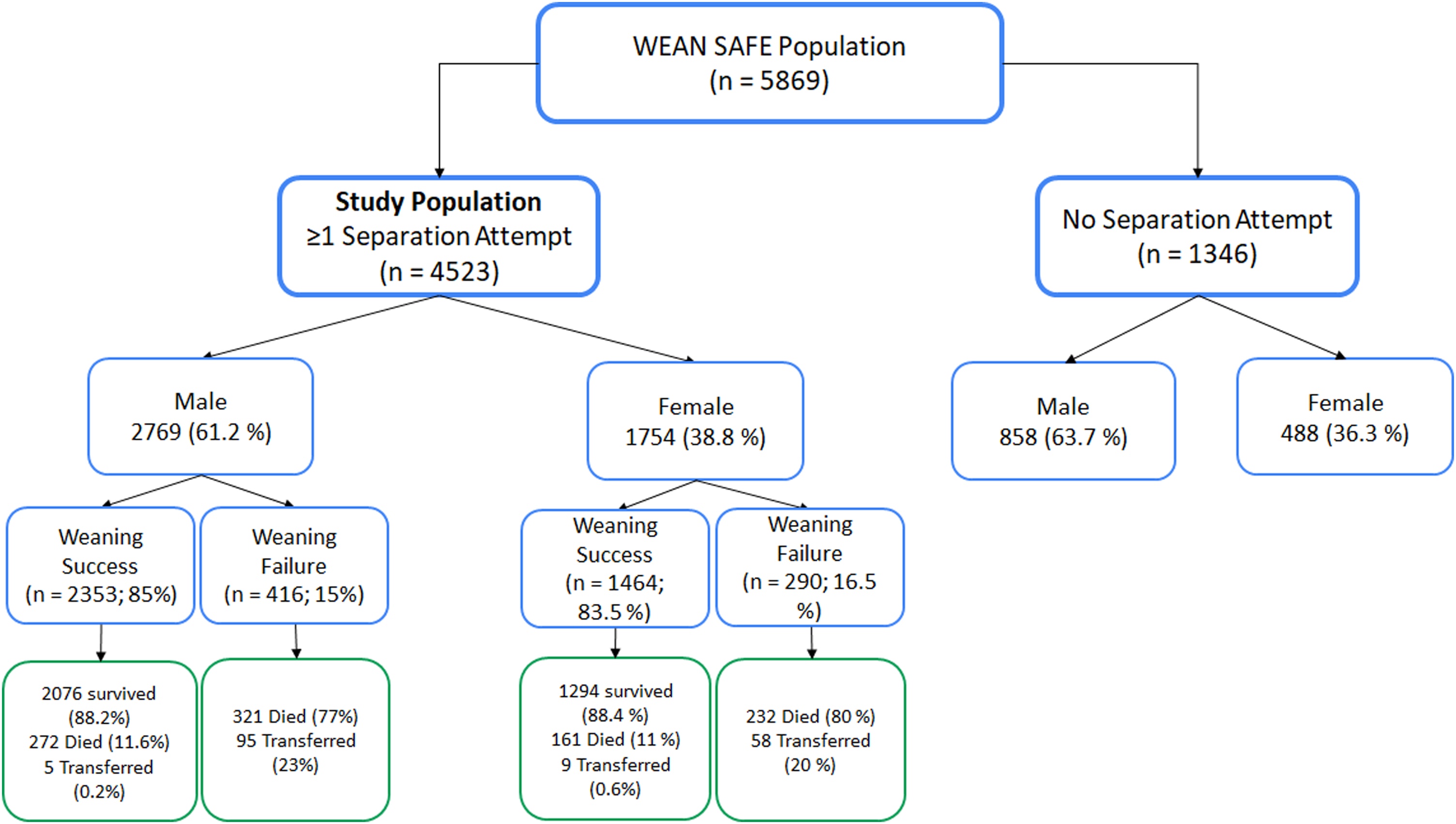
Flow chart for impact of male and female sex on the Weaning process and outcomes. Of note, patients ‘transferred’ were transferred from the study ICU to another facility prior to discharge and hence were lost to subsequent follow-up.

All patients admitted to a participating ICU aged >16 years, receiving invasive MV for two calendar days after intubation, and providing informed consent (where required), were included in the WEAN SAFE study. The study population for this analysis consisted of all patients undergoing at least 1 separation attempt [Fig. 1]. Patients transferred to other facilities before successful weaning were deemed lost to follow-up and their ICU and hospital outcomes were not collected. All data were recorded for each patient at the same time each day within participating ICUs, normally as close as possible to 10am each day.

### Data definitions

Our data definitions have been previously reported [1]. Briefly, initiation of weaning from invasive MV is defined as the time that the first separation attempt (SA) from the ventilator was performed [20]. A separation attempt is defined as a short period of either T-tube trial, low respiratory support (i.e., continuous positive airway pressure of ≤5 cm H_2_O, or pressure support ventilation with PEEP ≤ 5 cm H_2_O and pressure support ≤7 cm H_2_O), a short period of tracheostomy mask oxygenation, or a spontaneous breathing trial (SBT) as declared by the investigator, or extubation without an SBT. Weaning eligibility criteria comprised a fractional concentration of oxygen in inspired air (FiO2) less than 0·5, and positive end-expiratory pressure (PEEP) less than 10 cm H2O and receiving no or low doses of vasopressors (<0·2 μg/kg per min of norepinephrine or equivalent), and not receiving paralysing agents [1].

Delayed initiation of weaning is defined as a delay of >1 day from meeting weaning eligibility criteria to the first SA [1]. Increased weaning duration is defined as requiring >1 day to wean from invasive MV following the first SA. Comorbidities were defined as previously described (Supplemental Table S1) [21].

Weaning success is defined as extubation without death or reintubation within the following 7 days, or discharge from the study ICU without invasive MV within 7 days, whichever came first. For tracheostomised patients, weaning success is defined as spontaneous ventilation through tracheostomy without any MV during 7 consecutive days or discharged from the intensive care unit with unassisted breathing, whichever came first. We define weaning failure as those patients that entered the weaning process, but who either died, were transferred, or were still invasively ventilated at day 90.

Duration of invasive MV was calculated as the number of days between the date of intubation and the date of extubation in ICU (or death, if the patient died while receiving invasive MV). Survival was assessed at ICU discharge with follow-up extending up to 28 days, or at hospital discharge with follow-up extending up to 90 days. Information about the limitation of life-sustaining measures was also recorded.

### Study outcomes

The primary outcome of the study was successful weaning from invasive MV, and the primary objective was to investigate the impact of biologic sex on the likelihood of successful weaning from invasive MV. A key secondary objective was to understand the impact of height, age and frailty profile on the weaning process, as these differ significantly in women undergoing weaning compared to men. Other objectives included determination of the relationship between biologic sex and secondary outcomes of ventilatory management, survival to ICU and hospital discharge, decisions to limit life-sustaining interventions, and the risk factors for delayed and failed weaning and for survival in both sexes.

### Statistical analysis

Descriptive statistics were presented as proportions for categorical variables and as mean (standard deviation) or median (interquartile range) for continuous variables. Comparisons between male and female patients were conducted using the chi-square test for categorical variables and either the Wilcoxon rank-sum test or Student’s t-test for continuous variables, as appropriate.

Univariable and multivariable analyses were performed in the overall cohort and in the subgroup of sicker patients defined by a P/F ratio less than 200, at inclusion into the WEAN SAFE Study. The association of sex and height with time to weaning success, as well as time to ICU and hospital mortality, was examined using a multivariable Cox regression model. The proportional hazard (PH) assumption of Cox models was tested using the cox.zph() function in R. An ordinal multivariable logistic regression model was used to analyze weaning duration (0–1 days, 2−7 days, 7+ days), while delayed initiation of weaning and limitations on life-sustaining interventions were assessed using multivariable logistic regression models. An interaction between sex and height was tested for each of these outcomes but was then dropped from the final model if not statistically significant (at the 0.05 level). Each model was adjusted for potential confounders such as age, frailty, BMI, and height. Additionally, each model accounted for Lung Injury Indices on the first day of fulfilling weaning eligibility criteria (WEC), including PEEP, P/F ratio, respiratory rate, PIP minus PEEP, and the use of paralytic medications. Further adjustments were made for sedation levels and admission diagnoses, such as cardiac arrest, trauma, or non-traumatic neurological events. These variables were selected based on prior research on weaning outcomes in the ICU. Kaplan–Meier curves were generated to compare survival between male and female patients.

No assumptions were made for missing data; a complete case analysis was performed. As previous modelling of the outcomes in the primary analysis paper found no significant clustering by centre or country, we conducted all subsequent analyses using a parsimonious model, i.e. without random effects. We report odds ratios (OR) for the logistic models and hazard ratio (HR) for Cox regression models, along with 95% confidence intervals and p-values. All statistical analyses were performed using R software, version 4.4 (R Project for Statistical Computing, http://www.R-project.org). A two-tailed p-value of <0.05 was considered statistically significant.

## Results

Of 5,869 patients screened, 4,523 (80%) entered the weaning process and formed the study cohort (Fig. 1). The cohort comprised 1,754 women (38.8%) and 2,769 men (61.2%). Women were slightly older, were of shorter stature than men (Table 1). Women were more often admitted for medical reasons, particularly hypercapnic respiratory failure, while men more frequently presented with trauma or post-cardiac arrest. Comorbidity profiles were broadly similar, although women were less likely to have hepatic or neuromuscular disease (Table 1). In a sub-analysis, a total of 1,363 (30%) patients with more severe respiratory failure were selected based on a P/F ratio <200 at study entry.

**Table 1:** Demographics and outcome data in patients that entered the weaning process (n = 4523).

Characteristic	Men, N = 2,769	Women, N = 1,754	Missing data N (%)	p-value*
Age (years)	60 ± 17	62 ± 17	0 (0 %)	<0.001
Frailty Status (CFS Score >4, %)	560 (20%)	397 (23%)	30 (0.8%)	0.061
BMI (Kgs/m^2^)	27 ± 6	27 ± 8	158 (3.5 %)	0.965
Height (cm)	173 ± 8	160 ± 8	136 (3.0 %)	<0.001
Weight (kg)	80 ± 21	71 ± 21	83 (1.8 %)	<0.001
**ICU admission category**				
Medical	1,817 (66%)	1,210 (69%)	0 (0 %)	<0.001
Planned Surgery	235 (8%)	145 (8.3%)		
Trauma	306 (11%)	98 (5.6%)		
Urgent Surgery	411 (15%)	301 (17.1%)		
Cause(s) for ICU admission				
Hypoxemic respiratory failure	903 (33%)	597 (34%)	0 (0 %)	0.321
Sepsis	591 (21%)	412 (23%)		0.091
Hypercapnic respiratory failure	378 (14%)	284 (16%)		0.019
Non-traumatic neurologic event	373 (13%)	283 (16%)		0.013
Emergency surgery	392 (14%)	249 (14%)		0.970
Airway protection	359 (13%)	203 (12%)		0.167
Cardiac arrest	251 (9.1%)	115 (6.6%)		0.003
Comorbidities				
Respiratory	588 (21%)	387 (22%)	0 (0 %)	0.509
Cardiovascular	295 (11%)	206 (12%)	0 (0 %)	0.255
Liver	141 (5.1%)	58 (3.3%)	0 (0 %)	0.004
Kidney	286 (10%)	183 (10%)	0 (0 %)	0.910
Neuromuscular	706 (26%)	338 (19%)	26 (0.6 %)	<0.001
Immune Dysfunction	378 (14%)	259 (15%)	0 (0 %)	0.293
Diabetes	570 (21%)	401 (23%)	0 (0 %)	0.069
Hematologic malignancies	65 (2%)	40 (2%)	0 (0%)	0.884
Clinical Outcomes				
Total duration of invasive MV, days	6 (4, 12)	7 (4, 12)	153 (3.4%)	0.146
Length of ICU stay, days	11 (7, 18)	11 (7, 18)	154 (3.4%)	0.276
Length of hospital stay, days	23 (14, 39)	23 (14, 40)	213 (4.7%)	0.957
Limitation of life-sustaining interventions	459 (17%)	329 (19%)	0 (0 %)	0.060
ICU mortality	367 (14%)	266 (16%)	153 (3.4%)	0.073
Hospital mortality	593 (22%)	393 (23%)	167 (3.7%)	0.408

The data are n (%), mean ± SD, or median (Q1, Q3) where Q1 and Q3 represent 25th and 75th percentile, respectively. The p-value compares the two groups using Wilcoxon rank-sum test for continuous and Chi-squared test for categorical variables. For ICU admission category, a single global p-value (<0.01) indicates the overall distribution of categories differs between males and females.

### Respiratory failure severity

Women weaning from ventilation had significantly higher oxygenation (P/F ratios) at study inclusion, on the first day fulfilling weaning eligibility criteria, and at the time of the first separation attempt, compared to male patients (Table 2). Dynamic respiratory compliance was slightly higher in women than in men at study inclusion and the first day fulfilling weaning eligibility criteria (Table 2). There was small yet statistically significant differences in dynamic driving pressure (PIP minus PEEP) early during invasive ventilation, which became non-significant by the first separation attempt. Women consistently received lower PEEP levels at all time points compared to men (Table 2).

**Table 2:** Respiratory Failure Severity and Ventilatory support data in patients receiving controlled ventilation (n = 3056) at time of fulfilling Weaning Eligibility Criteria.

	Men, N = 1855 (68% Total)	Women, N = 1201 (70% Total)	Missing data N (%)	p-value
Tidal Volumes (ml/Kg IBW)				
At study Inclusion (Day 3−4)	7.7 ± 1.9	8.6 ± 2.0	87 (3%)	<0.001
First day of fulfilling WEC	7.4 ± 1.8	8.3 ± 2.0	98 (3%)	<0.001
At first SA	7.5 ± 1.9	8.4 ± 2.1	106 (3%)	<0.001
PEEP (cm H_2_O)				
At study Inclusion (Day 3−4)	7.0 (5.0, 10.0)	6.0 (5.0, 8.0)	28 (1%)	<0.001
First day fulfilling WEC	6.0 (5.0, 8.0)	5.0 (5.0, 8.0)	36 (1%)	0.013
At first SA	5.0 (5.0, 8.0)	5.0 (5.0, 7.0)	23 (1%)	0.039
Respiratory Rate (per minute)				
At study Inclusion (Day 3−4)	20.9 ± 5.5	20.5 ± 5.8	3 (0%)	0.020
First day fulfilling WEC	19.4 ± 5.3	19.6 ± 15.4	5 (0%)	0.065
At first SA	20.2 ± 6.0	20.1 ± 6.1	9 (0%)	0.762
P/F Ratio				
At study Inclusion (Day 3−4)	238.2 ± 97.2	249.5 ± 102.1	179 (6%)	0.007
First day of fulfilling WEC	271.1 ± 97.8	276.5 ± 100.0	10 (0%)	0.174
At first SA	277.5 ± 97.4	290.0 ± 104.9	5 (0%)	0.002
Peak Inspiratory Pressure				
At study Inclusion (Day 3−4)	24.0 (20.0, 29.0)	24.0 (20.0, 29.0)	35 (1%)	0.709
First day fulfilling WEC	21.0 (17.0, 25.0)	21.0 (17.0, 25.0)	44 (1%)	0.056
At first SA	18.0 (14.0, 23.0)	18.0 (14.0, 22.0)	47 (2%)	0.729
Dynamic Driving Pressure (PIP – PEEP)				
At study Inclusion (Day 3−4)	16.0 (13.0, 21.0)	17.0 (13.0, 22.0)	65 (2%)	0.044
First day fulfilling WEC	14.0 (11.0, 19.0)	15.0 (11.0, 19.0)	106 (3%)	0.009
At first SA	12.0 (8.0, 16.0)	12.0 (9.0, 16.0)	129 (4%)	0.565
Dynamic Compliance (Tidal volume/(PIP-PEEP))				
At study Inclusion (Day 3−4)	0.46 (0.34, 0.62)	0.51 (0.37, 0.68)	147 (5%)	<0.001
First day fulfilling WEC	0.50 (0.37, 0.71)	0.56 (0.40, 0.76)	194 (6%)	<0.001
At first SA	0.63 (0.43, 0.92)	0.69 (0.48, 1.01)	224 (7%)	<0.001
Sedation on the first day of fulfilling WEC				
Awake	401 (22%)	239 (20%)	11 (0%)	0.507
Moderate sedation	772 (42%)	511 (43%)		
Deep sedation	674 (37%)	448 (37%)		

The data are n (%), or mean ± SD, or median (Q1, Q3) where Q1 and Q3 represent 25th and 75th percentile, respectively. The p-value compares the two groups using Wilcoxon rank-sum test for continuous and Chi-squared test for categorical variables. Abbreviations: SA, separation attempt; WEC, weaning eligibility criteria.

### Ventilatory support

At the time of fulfilling weaning eligibility criteria, fewer men (1855, 68%) than women (1201, 70%) were receiving controlled ventilation, while more men (867, 32%) than women (526, 30%) were receiving assisted ventilation (p = 0.03). Women on controlled ventilation were subjected to higher driving pressures and received higher tidal volumes and lower PEEP compared to men (Table 2). Women on assisted ventilation were subjected to higher set inspiratory pressures than men, resulting in higher tidal volumes, and received lower PEEP, than men (Table 3). Tidal volumes decreased as a function of height in both sexes, an effect more pronounced in women given their shorter stature (Supplementary Fig. S1A-C, Table 1).

**Table 3:** Respiratory Failure Severity and Ventilatory support data in patients receiving assisted ventilation (n = 1393) at time of fulfilling Weaning Eligibility Criteria.

	Men, N = 867 (32% Total)	Women, N = 526 (30% Total)	Missing data N (%)	p-value
Tidal Volumes (ml/Kg IBW)				
At study Inclusion (Day 3−4)	8.2 ± 2.3	9 ± 2.3	133 (10%)	<0.001
First day of fulfilling WEC	7.7 ± 2.2	8.3 ± 2.1	147 (11%)	<0.001
At first SA	7.6 ± 2.3	8.1 ± 2.2	128 (9%)	<0.001
PEEP (cm H_2_O)				
At study Inclusion (Day 3−4)	6.0 (5.0, 8.0)	6.0 (5.0, 8.0)	38 (3%)	0.142
First day fulfilling WEC	5.0 (5.0, 8.0)	5.0 (5.0, 7.0)	60 (4%)	0.286
At first SA	5.0 (5.0, 7.0)	5.0 (5.0, 6.0)	33 (2%)	0.524
Respiratory Rate (per minute)				
At study Inclusion (Day 3−4)	21.1 ± 6.5	21.0 ± 6.5	8 (1%)	0.955
First day fulfilling WEC	18.9 ± 6.2	19.0 ± 6.4	14 (1%)	0.962
At first SA	19.5 ± 6.4	20.3 ± 6.7	11 (1%)	0.023
P/F Ratio				
At study Inclusion (Day 3−4)	260.2 ± 97.2	275.0 ± 101.6	133 (10%)	0.015
First day of fulfilling WEC	277.4 ± 94.7	292.4 ± 99.8	14 (1%)	0.009
At first SA	285.7 ± 98.5	298.1 ± 104.1	10 (1%)	0.046
Peak Inspiratory Pressure (PIP)				
At study Inclusion (Day 3−4)	16.0 (13.0, 20.0)	17.0 (13.0, 21.0)	47 (3%)	0.256
First day fulfilling WEC	15.0 (12.0, 18.8)	16.0 (13.0, 19.0)	57 (4%)	0.046
At first SA	14.0 (11.0, 18.0)	15.0 (11.0, 18.0)	46 (3%)	0.185
Set Inspiratory Pressure (PIP – PEEP)				
At study Inclusion (Day 3−4)	10.0 (7.0, 14.0)	11.0 (8.0, 14.5)	96 (7%)	0.035
First day fulfilling WEC	10.0 (6.0, 12.0)	10.0 (7.0, 13.0)	130 (9%)	0.023
At first SA	8.5 (6.0, 11.0)	9.0 (6.0, 12.0)	121 (9%)	0.057
Sedation on the first day of fulfilling WEC				
Awake	284 (33%)	179 (34%)	19 (1%)	0.135
Moderate sedation	419 (49%)	230 (44%)		
Deep sedation	151 (18%)	111 (21%)		

The data are n (%), or mean ± SD, or median (Q1, Q3) where Q1 and Q3 represent 25th and 75th percentile, respectively. The p-value compares the two groups using Wilcoxon rank-sum test for continuous and Chi-squared test for categorical variables. Abbreviations: SA, separation attempt; WEC, weaning eligibility criteria.

**Fig. 2:**
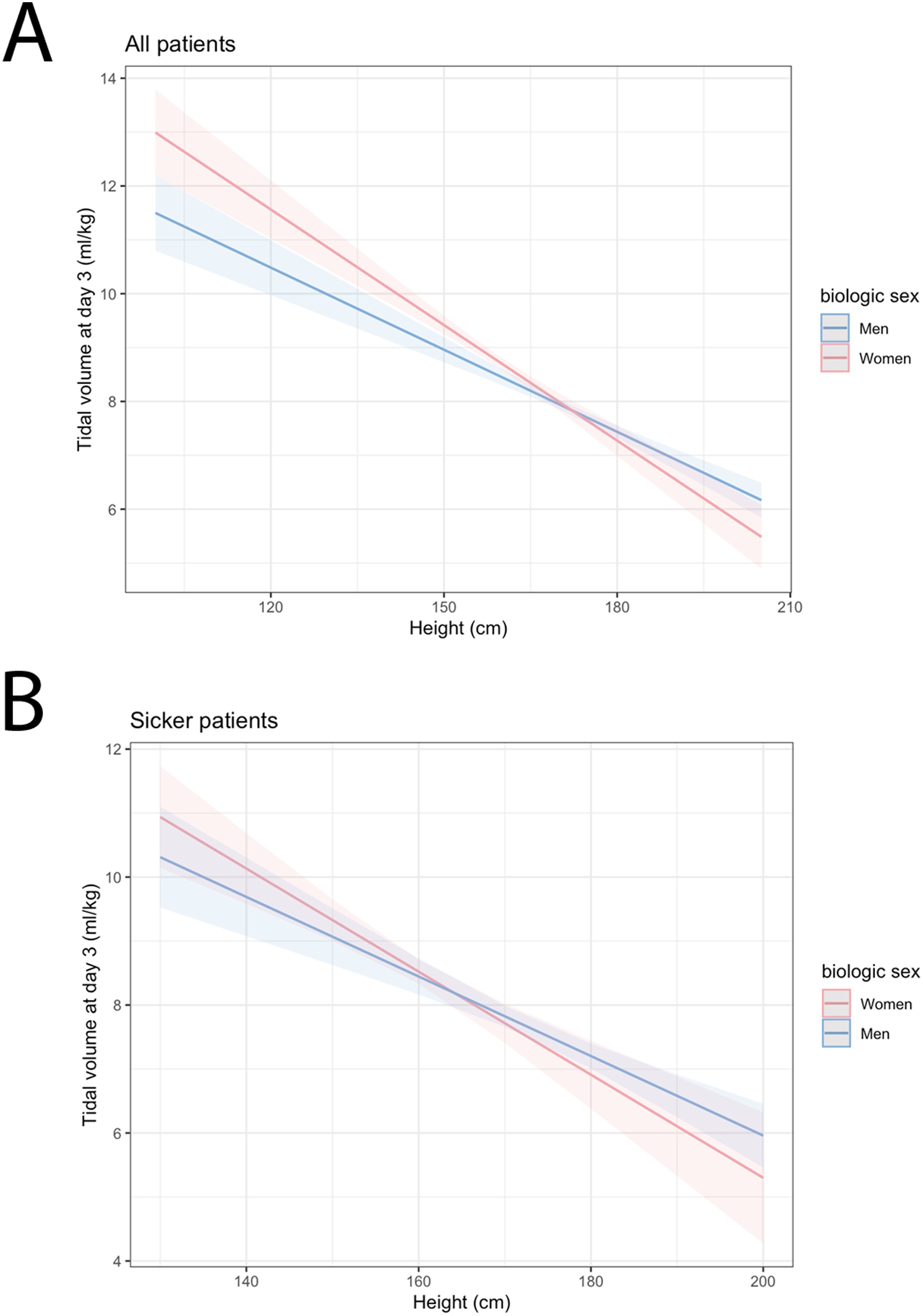
Association between height and tidal volumes at day 3 stratified by sex. Fitted tidal volumes at day 3 versus height stratified by sex for all patients (***Panel A***) and patients with P/F ratio <200 (***Panel B***). Lines are multivariable linear model fits with 95% confidence intervals.

In the multivariable analysis, greater height (β = −0.05, p < 0.001) was independently associated with lower tidal volumes, female sex (β = 3.56, p = 0.008) was independently associated with higher tidal volumes, and the interaction term between height and sex (β = −0.02, p = 0.012) was also significant (Table 4). This indicates that women had higher baseline tidal volumes than men and had a steeper decline in tidal volumes with increasing height (Fig. 2A). In addition, older age was correlated with shorter stature (−0.15, p < 0.001), further underlining the inter-relationships between sex, height and age in this cohort.

**Table 4:** Multivariable regression models of higher tidal volume (VT/PBW) at day 3 (n = 4523).

Variable	Coefficient	Lower 95% CI	Upper 95% CI	p-value
Female Sex	3.56	0.91	6.20	0.008
Height (cm)	−0.05	−0.06	−0.04	<0.001
Sex* Height	−0.02	−0.04	−0.00	0.012
Age	0.01	0.01	0.01	<0.001
Planned Surgery	0.24	0.01	0.46	0.038
Trauma	0.21	−0.02	0.44	0.076
Urgent Surgery	0.27	0.10	0.44	0.002
Comorbidities				
Respiratory	−0.05	−0.2	0.11	0.559
Cardiovascular	−0.13	−0.33	0.07	0.212
Liver	0.51	0.20	0.82	0.001
Kidney	−0.02	−0.23	0.19	0.826
Neuromuscular	−0.14	−0.29	0.01	0.062
Immune Dysfunction	−0.13	−0.30	0.05	0.166
Diabetes	0.10	−0.05	0.26	0.186
Lung Injury Indices				
P/F ratio at day 3	0.00	0.00	0.00	0.055

In the more severe respiratory failure subgroup, greater height remained independently associated with lower tidal volumes (β = −0.06, p < 0.001; Supplementary Table S2), whereas biologic sex was not associated with tidal volume delivered, and the sex–height interaction was not significant (Fig. 2B).

### Initiation of weaning

The frequency of moderate or deep sedation at the time of weaning eligibility were similar in women and men (Table 2, Table 3). The incidence of delayed initiation of weaning did not differ significantly between women and men (49% vs 46%, p = 0.07; Table 5). In univariable analysis, neither sex nor height was significantly associated with delayed weaning initiation (Supplemental Table S2). Multivariable analysis showed that sex and height were not independent predictors, while age, frailty, higher driving pressure, deep sedation, and neuromuscular blockade were associated with delayed weaning initiation (Table 6).

**Table 5:** Weaning Milestones and outcome data in patients that entered the weaning process (n = 4523).

Characteristic	Men, N = 2,769	Women, N = 1,754	Missing data N (%)	p-value*
Delayed initiation of weaning	1,278 (46%)	858 (49%)	0 (0%)	0.070
Number Separation Attempts				
1	1,345 (49%)	832 (47%)	0 (0%)	0.712
2	778 (28%)	497 (28%)		
≥3	646 (23%)	425 (24%)		
First Separation Attempt Type				
Direct Extubation	561 (20%)	364 (21%)	0 (0%)	0.919
SBT	1,828 (66%)	1,149 (66%)		
PSV on trach	380 (14%)	241 (14%)		
Number of Extubations				
0	525 (19%)	344 (20%)	0 (0%)	0.379
1	2,059 (74%)	1,304 (74%)		
2	154 (6%)	95 (5%)		
≥3	31 (1%)	11 (1%)		
Reintubation				
Reintubation at 48 h (n = 352 patients)	218 (8%)	134 (8%)	0 (0%)	0.891
Reintubation by day 7 (n = 499 patients)	310 (11%)	189 (11%)	0 (0%)	0.725
Tracheostomy				
At any time (n = 967 patients)	587 (21%)	380 (22%)	0 (0%)	0.710
Already present at Day 3 (n = 137 patients)	95 (3%)	42 (2%)	0 (0%)	0.028
Inserted after at least 1 SA (n = 346 patients)	207 (8%)	139 (8%)	0 (0%)	0.677
Initial level of Post Extubation Respiratory Support (n = 3654 patients)				
Non-Invasive Ventilation	352 (17%)	225 (17%)	301 (7%)	0.964
High Flow Oxygen	368 (18%)	228 (18%)		
Low flow Oxygen	1,335 (65%)	845 (65%)		
Highest level of Post Extubation Respiratory Support (n = 3654 patients)				
Non-Invasive Ventilation	471 (23%)	291 (23%)	301 (7%)	0.939
High Flow Oxygen	360 (17%)	227 (17%)		
Low flow Oxygen	1,224 (60%)	780 (60%)		
Weaning Duration (successfully weaned)				
Short Wean (≤1 day)	1,807 (77%)	1,120 (77%)	0 (0%)	0.723
Intermediate Wean (2−7 d)	286 (12%)	171 (12%)		
Prolonged Wean (>7 days)	260 (11%)	173 (11%)		
Weaning outcomes				
Failed weaned	416 (15%)	290 (17%)	0 (0%)	0.173
Successfully Weaned	2,353 (85%)	1,464 (83%)		

The data is n (%). The p-value compares the two groups using Wilcoxon rank-sum test for continuous and Chi-squared test for categorical variables. Abbreviations: SBT, spontaneous breathing trial; PSC, pressure support ventilation.

**Table 6:** Multivariable models of association between female sex and height on weaning and clinical outcomes (n = 4523).

		Lower 95% CI	Upper 95% CI	p-value
Delayed Weaning initiation^1^^,^^2^	Adjusted Odds Ratio			
Female Sex	1.030	0.875	1.211	0.723
Height	0.995	0.987	1.003	0.195

Weaning Duration Category^1^^,^^3^	Adjusted Odds Ratio			
Female Sex	1.045	0.856	1.275	0.666
Height	1.004	0.994	1.014	0.455

Weaning Success^1^^,^^4^	Adjusted Hazard Ratio			
Female Sex	0.922	0.848	1.002	0.055
Height	0.999	0.995	1.003	0.623

ICU Mortality^1^^,^^4^	Adjusted Hazard Ratio			
Female Sex	1.115	0.881	1.411	0.364
Height	1.002	0.990	1.014	0.759

Hospital Mortality^1^^,^^4^	Adjusted Hazard Ratio			
Female Sex	1.083	0.912	1.286	0.364
Height	1.000	0.991	1.009	0.951

Limit Life Sustaining Measures^1^^,^^2^	Adjusted Odds Ratio			
Female Sex	1.194	0.957	1.489	0.117
Height	1.004	0.993	1.015	0.487

1 Each analysis is adjusted for demographics (Age, Frailty status), lung injury indices (P/F ratio, Respiratory Rate, Use of Neuromuscular blockade), Sedation levels and reasons for ICU Admission (Cardiac arrest, Trauma, Neurologic (non-trauma)).

2 Multivariable Logistic Regression Model.

3 Multivariable Ordinal Logistic regression model.

4 Multivariable Cox Regression Model.

In the subgroup of patients with more severe respiratory failure (P/F ratio less than 200), sex and height were not significantly associated with delayed initiation of weaning (adjusted OR 0.91, 95% CI 0.67–1.24), weaning duration (adjusted OR 0.99, 95% CI 0.69–1.44), or weaning success (adjusted HR 0.96, 95% CI 0.81–1.13) in the multivariable analysis (Supplemental Tables S3–S5).

### Weaning process and milestones

**Fig. 3:**
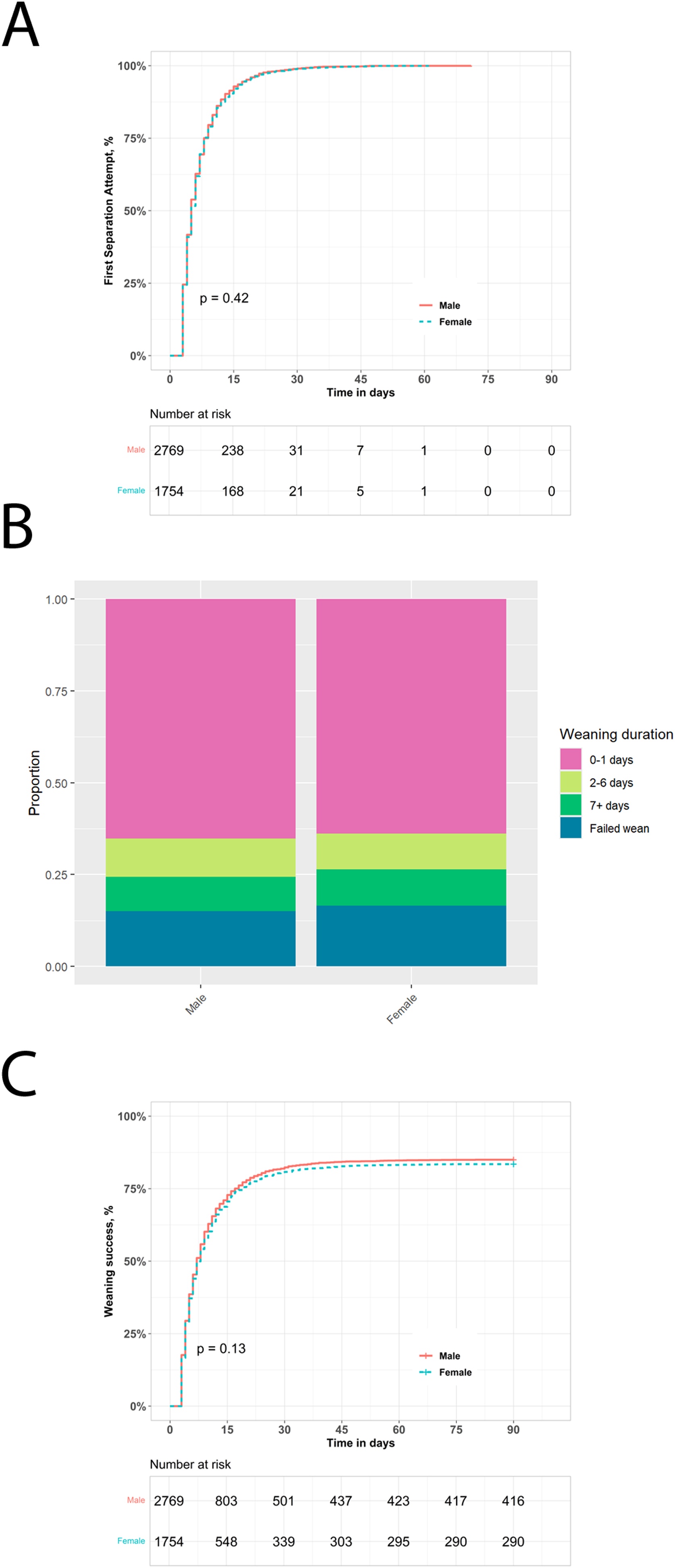
Impact of biologic sex on weaning from invasive ventilation. Kaplan-Meier analysis of impact of biologic sex on likelihood of entering the weaning process (***Panel A***). Stacked bar chart of impact of biologic sex on weaning outcomes in the study population (***Panel B***). Kaplan–Meier analysis of impact of biologic sex on weaning success probability over time to Day 90 (***Panel C***).

Once initiated, progression through the weaning process was largely similar for women and men. The number of separation and extubation attempts, reintubation rates, tracheostomy use, and post-extubation respiratory support strategies showed no sex-based differences (Fig. 3A–C, Table 5). The distribution of weaning duration (short, intermediate, prolonged) was also nearly identical between groups (Fig. 3B). In multivariable ordinal analysis, neither biologic sex nor height were independently associated with a longer weaning duration (Table 6), and there was no interaction between height and sex in the adjusted model. Post-extubation respiratory support strategies were similar in men and women: most patients in both groups received low-flow oxygen therapy after extubation, with smaller subsets receiving high-flow oxygen or non-invasive ventilation as initial or highest support, without significant sex-based differences (Table 5).

### Weaning outcomes

Weaning success rate did not differ in women compared to men (83% vs 85%, p = 0.173) (Fig. 3C**,** Table 5). Female sex was significantly associated with weaning failure in univariable analysis, with women having a 7% lower chance of successful weaning compared to men (HR = 0.93, 95% CI: 0.87−0.99, p = 0.026, Supplementary Table S1). Multivariable analysis showed no independent association between biologic sex and weaning success (Table 6) after further adjustment for additional covariates, such as height, age, frailty profile and sedation. This can be explained by potential confounding between sex, age and height, given that women in our cohort were significantly older and shorter than men on average (62 ± 17 vs 60 ± 17, p < 0.001; 160 ± 8 vs 173 ± 8, p < 0.001; Table 1). In the subgroup of patients with a P/F ratio less than 200, sex and height were not significantly associated with weaning success and were not independent predictors in the multivariable analysis.

### Clinical outcomes

**Fig. 4:**
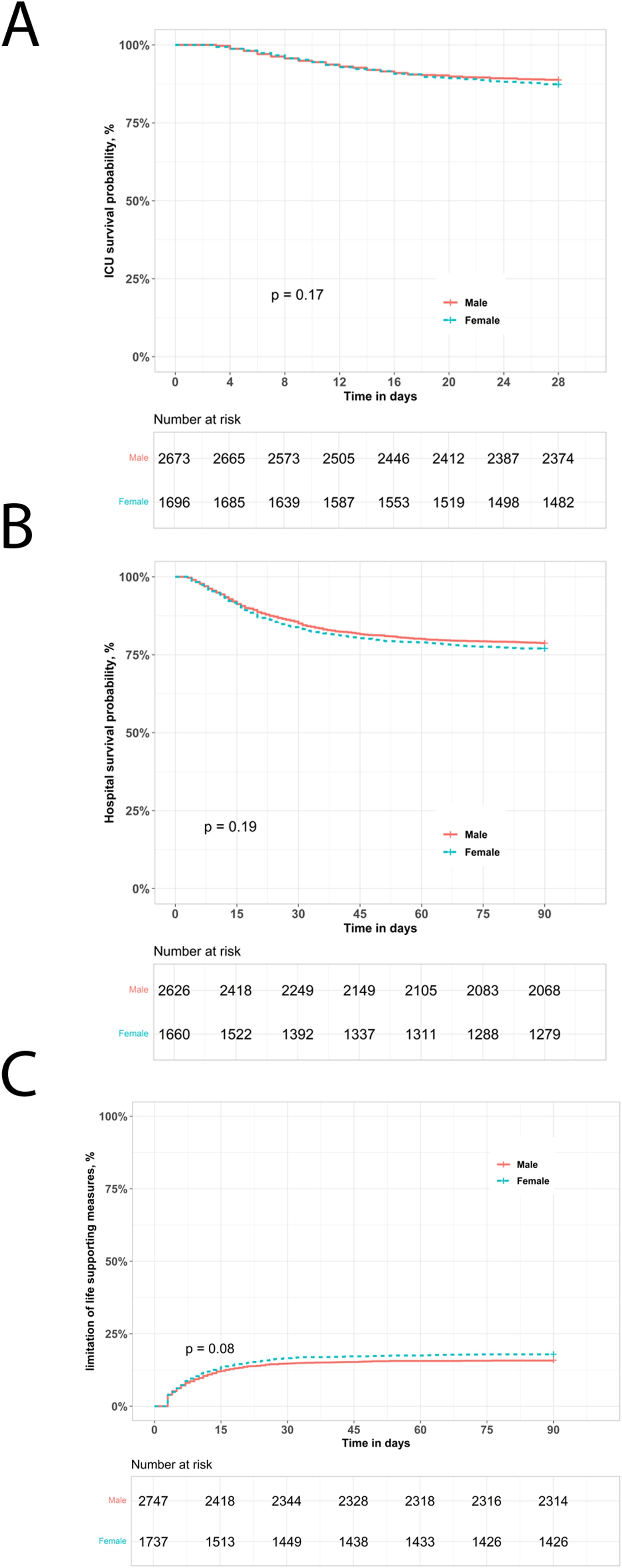
Impact of biologic sex on clinical outcomes in patients weaning from invasive ventilation. Kaplan–Meier analysis of impact of biologic sex on ICU survival probability over time to Day 28 (***Panel A***). Kaplan–Meier analysis of impact of biologic sex on hospital survival probability over time to Day 90 (***Panel B***). Kaplan–Meier plot of impact of biologic sex on probability of limitation of life supporting measures (***Panel C***).

The duration of invasive ventilation, ICU and hospital stay lengths, and survival were similar for women and men (Fig. 4, Table 1, Table 5). Multivariable analyses confirmed that neither sex nor patient height were an independent predictor of ICU or hospital mortality, or of limitations on life-sustaining interventions (Table 6). In contrast, height showed a significant association with hospital mortality in univariable analysis (HR = 0.99, 95% CI: 0.98–1.00, p = 0.003), while this association was attenuated after adjusting for age. This is likely because there was a weak but significant correlation between age and height (-0.15, p < 0.001) in our cohort. Likewise, height (OR = 0.99, 95% CI:0.98–1.00, p = 0.027) was significantly associated with limit life-sustaining interventions in the univariable analysis, whereas age, frailty and trauma were confounders which attenuated this significant association in the multivariable analysis.

In the subgroup of patients with a P/F ratio less than 200, height was still significantly associated with hospital mortality (HR = 0.99, 95% CI: 0.98–1.00, p = 0.025) and limitation of life-sustaining interventions (OR = 0.98, 95% CI: 0.97–1.00, p = 0.018) in univariable analyses (Supplementary Table S3). However, height was not an independent predictor of hospital mortality and limit life-sustaining interventions in the multivariable analysis due to potential confounding.

### Discussion

The process of weaning from invasive mechanical ventilation represents a critical juncture in the care of critically ill patients, influencing morbidity, mortality, and healthcare resource utilization. Previous studies have highlighted sex-based disparities in various aspects of critical care, including vital organ support [22], ICU admission rates [23], mortality [22,24], and ventilatory management [12]. However, the impact of biologic sex on the weaning process itself is less clear, with some studies reporting poorer weaning outcomes for men [15], while others report no sex-based differences in weaning outcomes [25,26].

Our study, a predefined secondary analysis of the WEAN-SAFE dataset, found that females were slightly older and of notably shorter stature, and had less severe hypoxia than males. Despite having less severe respiratory failure, women received less protective ventilation. Women on controlled ventilation received higher driving pressures, while women on assisted ventilation received higher inspiratory pressures than men, resulting in higher tidal volume ventilation in women. Women were less likely to successfully wean from ventilation, potentially because of their shorter stature, and their older age, compared to men.

### Ventilatory management

Women appeared to have less severe respiratory failure, as evidenced by their consistently higher oxygenation levels, and better dynamic lung compliance at key timepoints during the study. Despite this, at the time of fulfilling weaning eligibility criteria, fewer women were receiving assisted ventilation compared to men. Of concern, women received less protective ventilation than their male counterparts. In controlled ventilation modes, women were subjected to higher driving pressures and received higher tidal volumes and lower PEEP compared to men. In assisted ventilation modes, women were subjected to higher set inspiratory pressures than men, resulting in higher tidal volumes, suggesting they were ‘over-assisted’ [27], which is associated with higher risk of ventilator induced lung injury [28], greater diaphragm dysfunction and atrophy [29], and greater patient-ventilator dyssynchrony [30], and weaning failure [28,29].

Multivariable analyses confirmed independent associations of both female sex and shorter stature with higher tidal volumes, with a significant interaction indicating that shorter women are at particular risk. These results are consistent with prior evidence showing that shorter individuals (disproportionately women) often receive larger tidal volumes relative to their lung size, potentially increasing the risk of ventilator-induced lung injury (VILI) [[12], [13], [14],^31^]. Short stature has been demonstrated to be an independent risk factor for mortality in ARDS, potentially mediated via greater ventilatory inefficiency [32].

The relationship between these factors is likely explained by the fact that tidal volume is conventionally ‘dosed’ according to ideal body weight, which in turn is a function of height and biologic sex. Our finding that older age was correlated with shorter stature further underlining the inter-relationships between sex, height and age in this cohort. These findings raise concerns about systemic biases in ventilatory management and underline the need for more care in setting tidal volumes in older, shorter female patients.

### Impact on weaning duration and milestones

We found no evidence that sex independently influenced the initiation or trajectory of weaning. Instead, delays in weaning were strongly associated with factors such as advanced age, frailty, higher driving pressures, deep sedation, and use of neuromuscular blockade. These observations underscore the dominance of clinical severity and management practices over sex-specific factors in determining the timing of weaning initiation.

The subsequent progression through the weaning process including separation and extubation attempts, reintubation, tracheostomy, and post-extubation support was strikingly similar between women and men. This contrasts with findings from Thille and colleagues, who reported male sex as a predictor of extubation failure in a high-risk cohort [15], suggesting that differences in the study population may account for these differences. Collectively, our findings suggest a largely uniform weaning trajectory once the process is initiated.

### Impact on weaning outcomes

In the WEAN SAFE primary paper [1], higher P/F ratio at the time of first separation attempt was associated with greater likelihood of weaning success. In this analysis, we demonstrate that women had higher P/F ratios at all key stages of the weaning process. Despite this, women were less likely to successfully wean compared to men, a finding of significant concern. Following adjustment for factors such as height, age, sedation levels and frailty profile, female sex was no longer associated with reduced likelihood of weaning success. This suggests that women’s shorter stature, older age profile and increased frailty profile may explain the higher risk of weaning failure seen in women. The adverse ventilatory management seen in women may have also contributed to their lack of weaning success.

### Impact on clinical outcomes

Regarding overall clinical outcomes, our study found no significant sex-based differences in ICU or hospital mortality rates, a finding confirmed in both univariable and multivariable adjusted analyses. Given that higher P/F ratios are generally associated with better ICU and hospital survival rates [33], the numerically higher ICU and hospital mortality rates in women suggest a potential concern.

The likelihood of receiving limitations on life-sustaining interventions was lower in women with more severe respiratory failure. When adjusted for factors such as height, age, sedation levels and frailty profile, female sex was no longer associated with increased likelihood of receiving limitations on life-sustaining interventions.

Moreover, our study found height was significantly associated with hospital mortality and limitations of life-sustaining interventions in the univariable analyses, which suggests shorter patients were more likely to have higher hospital mortality and to receive less aggressive interventions. However, these significant associations were attenuated in the multivariable analyses after adjustment for confounding variables.

Taken together, our finding may help explain findings in prior studies suggesting critically ill women may receive less aggressive interventions [5,7], and have poorer clinical outcomes. In our cohort, women were somewhat older and tended to be frailer and were of shorter stature. Older age profile and greater frailty are both known to influence outcomes and were indeed associated with poorer ICU and hospital survival and decisions to limit life-sustaining measures in the wider WEAN SAFE population [4]. Shorter stature has been repeatedly demonstrated to be associated with poorer outcomes in critical care [32]. Our analysis suggests that the clinical course and outcomes for men and women, once they enter the weaning process, appear to be similar, despite initial differences in critical illness presentation and ventilatory management, once differences in age profile, frailty, and stature are taken into account.

### Impact of respiratory failure severity

An important consideration when interpreting the observed sex-based differences in ventilatory management is the potential confounding effect of respiratory failure severity. In the overall cohort, women consistently had higher P/F ratios at study inclusion, at the time of fulfilling weaning eligibility criteria, and at the first separation attempt, indicating less severe hypoxaemia compared with men. In the subgroup of patients with more severe acute respiratory failure, as defined by a P/F ratio of <200 at study inclusion, the sex-based differences observed in the overall cohort were no longer evident. Ventilatory management, including tidal volume delivery, and key weaning outcomes were similar in women and men. While height remained associated with tidal volume, neither biologic sex nor the sex–height interaction influenced ventilatory settings or weaning success in this subgroup. This contrasts with the overall cohort, in which women—particularly those of shorter stature—were more likely to receive higher tidal volumes and ventilatory over-assistance.

These findings suggests that ventilatory and weaning practices may be better standardized and protocolised weaning strategies in patients with more severe respiratory failure. Importantly, outcomes in this subgroup appeared to be driven primarily by age, frailty, and severity of illness rather than by biologic sex. As lung-protective ventilation strategies are more consistently applied in patients with more severe respiratory failure, some of the observed differences in tidal volume delivery and ventilatory support may reflect differences in illness severity rather than biologic sex per se. Furthermore, efforts to reduce sex-based disparities in mechanical ventilation should focus on patients with less severe respiratory failure, where clinical discretion is greater and adherence to severity-driven protocols may be less consistent.

### Strengths and limitations

A major strength of our study is its large, multinational dataset, which includes ICUs across 50 countries, enhancing the generalizability of our findings. Our study investigates the impact of weaning from invasive mechanical ventilation in both men and women within a globally diverse patient cohort. Additionally, the structured data collection methodology of the WEAN-SAFE study allowed for standardized definitions of weaning parameters, minimizing potential bias in outcome assessment. The use of multivariable models enabled us to adjust for key confounders, providing robust estimates of the association between biologic sex and weaning outcomes. To further ensure data integrity, we implemented a rigorous data quality control program, as previously described [1].

However, several limitations should be acknowledged. We do not know to what extent height was measured or estimated in patients enrolled in WEAN SAFE. Height is often overestimated in clinical practice in the ICU, especially in shorter patients [34]. This suggests that shorter – predominantly female – patients might have received even higher tidal volumes than they would have if their height was measured. Although all raw data were directly entered into the electronic case report form, the interpretation of source data was carried out by on-site clinicians, which may have introduced variability. While participating hospitals represented a range of care levels and geographic regions, our convenience sampling approach may still have introduced selection bias. Additionally, our definitions of weaning success and failure do not clearly differentiate between weaning failure and extubation failure. Another limitation is our assumption that patients discharged before day 90 were still alive at that time. Furthermore, a small subset of patients (4%) was lost to follow-up due to transfer before their first separation attempt.

As with all observational studies, our findings cannot establish causality. While we adjusted for multiple confounders, unmeasured variables—such as clinician decision-making biases or patient-specific respiratory mechanics—may have influenced weaning outcomes. Our results include many different analyses, and so there is an associated risk of a type 1 error. Lastly, while we assessed ICU and hospital mortality, longer-term functional and quality-of-life outcomes were not evaluated, limiting our ability to determine the full impact of sex on post-ICU recovery.

## Conclusions

Women weaning from ventilation were older and tended to be frailer and were of shorter stature. Women received higher tidal volume ventilation and were more frequently subjected to ventilatory over-assistance, underscoring a persistent challenge in lung-protective ventilation strategies in women. While women were less likely to wean successfully from invasive ventilation, when adjusted for height and age, female sex *per se* was not independently associated with failed weaning from invasive ventilation.

## CRediT authorship contribution statement

Concept and design: AS, MD, PN, JGL.

Acquisition, analysis, or interpretation of data: All Authors.

Drafting of the manuscript: RC, KL, AS, JGL.

Critical revision of the manuscript for important intellectual content: All Authors.

Statistical analysis: OK, YZ, AS, JGL.

Administrative, technical, or material support: N/A.

Supervision: AS, JGL.

## Consent for publication

Not applicable.

## Funding

Jointly funded by the 10.13039/501100013347European Society of Intensive Care Medicine and the WEAN SAFE Clinical Research Collaboration from the European Respiratory Society. KL was funded by Health Research Board Summer Student Scholarships. AJS & YZ were funded by Research Ireland grant 19/FFP/7002. The funders had no direct involvement in the conduct of the study or preparation of the manuscript.

## Ethics approval and consent to participate

All participating ICUs obtained ethics committee approval and obtained either patient consent or ethics committee waiver of consent in the WEAN SAFE study. The WEAN SAFE study was registered at Clinicaltrials.gov, number NCT03255109.

## Availability of data and material

The data in this manuscript are owned by the individual contributing institutions of the WEAN SAFE investigators. Requests for data should be made to the WEAN SAFE Executive Committee, by way of email to the corresponding author. Any data provided will consist of de-identified participant with data dictionary, be restricted to the data presented in this paper, and be subject to a data sharing agreement.

## Declaration of competing interest

RC, KL, OK, ER, YZ, BM, TP, and AS have no competing interests. LH reports grants from Liberate Medical and InflaRx to his institution, honoraria from Getinge. GB reports a grant from Drager to his institution, consulting fees from Flowmeter, payments/honoraria from Drager and Getinge, and stock options in Dico technologies. LB reports grants from Medtronic, Drager and Stimity to his institution, honoraria and equipment received from Fisher Paykel. MD reports receiving fees from Fisher & Paykel and Lungpacer Medical Inc. PN reports research lab received grants/research equipment from Drager, Mindray, Intersurgical SPA, and Gilead. PN receives royalties from Intersurgical SPA for the Helmet Next invention. He also received speaking fees from Getinge, Intersurgical SPA, Gilead, MSD, GSK, Shionogi, Fisher & Paykel, Philips and Drager. JGL reports grants from Research Ireland and Health Research Board Ireland to his institution, and consulting fees from Cellenkos.

## Figures and Tables

**Figure:**
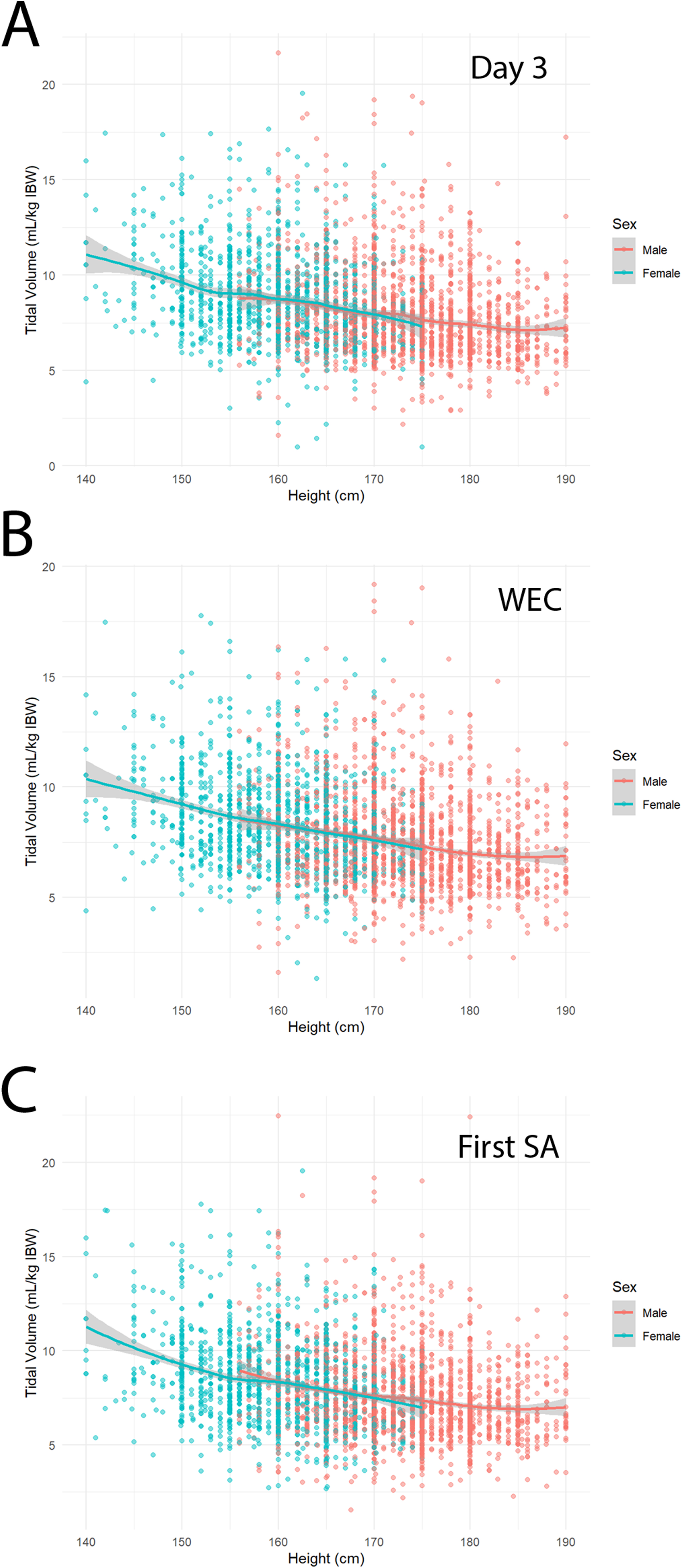


## References

[1] Pham T., Heunks L., Bellani G., Madotto F., Aragao I., Beduneau G., Investigators WS (2023). Weaning from mechanical ventilation in intensive care units across 50 countries (WEAN SAFE): a multicentre, prospective, observational cohort study. Lancet Respir Med.

[2] Epstein S.K., Ciubotaru R.L. (1998). Independent effects of etiology of failure and time to reintubation on outcome for patients failing extubation. Am J Respir Crit Care Med.

[3] Thille A.W., Richard J.C., Brochard L. (2013). The decision to extubate in the intensive care unit. Am J Respir Crit Care Med.

[4] Laffey C.M., Sheerin R., Khazaei O., McNicholas B.A., Pham T., Heunks L. (2025). Impact of frailty and older age on weaning from invasive ventilation: a secondary analysis of the WEAN SAFE study. Ann Intensive Care.

[5] Modra L.J., Higgins A.M., Abeygunawardana V.S., Vithanage R.N., Bailey M.J., Bellomo R. (2022). Sex differences in treatment of adult intensive care patients: a systematic review and meta-analysis. Crit Care Med.

[6] Todorov A., Kaufmann F., Arslani K., Haider A., Bengs S., Goliasch G., Swiss Society of Intensive Care M (2021). Gender differences in the provision of intensive care: a Bayesian approach. Intensive Care Med.

[7] Fowler R.A., Sabur N., Li P., Juurlink D.N., Pinto R., Hladunewich M.A. (2007). Sex-and age-based differences in the delivery and outcomes of critical care. CMAJ.

[8] Merdji H., Long M.T., Ostermann M., Herridge M., Myatra S.N., De Rosa S. (2023). Sex and gender differences in intensive care medicine. Intensive Care Med.

[9] Valentin A., Jordan B., Lang T., Hiesmayr M., Metnitz P.G. (2003). Gender-related differences in intensive care: a multiple-center cohort study of therapeutic interventions and outcome in critically ill patients. Crit Care Med.

[10] Blecha S., Zeman F., Specht S., Lydia Pfefferle A., Placek S., Karagiannidis C. (2021). Invasiveness of treatment is gender dependent in intensive care: results from a retrospective analysis of 26,711 cases. Anesth Analg.

[11] Zettersten E., Jaderling G., Bell M., Larsson E. (2020). Sex and gender aspects on intensive care. A cohort study. J Crit Care.

[12] McNicholas B.A., Madotto F., Pham T., Rezoagli E., Masterson C.H., Horie S., Investigators LS, the ETG (2019). Demographics, management and outcome of females and males with acute respiratory distress syndrome in the LUNG SAFE prospective cohort study. Eur Respir J.

[13] Han S., Martin G.S., Maloney J.P., Shanholtz C., Barnes K.C., Murray S. (2011). Short women with severe sepsis-related acute lung injury receive lung protective ventilation less frequently: an observational cohort study. Crit Care.

[14] Walkey A.J., Wiener R.S. (2012). Risk factors for underuse of lung-protective ventilation in acute lung injury. J Crit Care.

[15] Thille A.W., Boissier F., Coudroy R., Le Pape S., Arrive F., Marchasson L. (2023). Sex difference in the risk of extubation failure in ICUs. Ann Intensive Care.

[16] Townsend E.A., Miller V.M., Prakash Y.S. (2012). Sex differences and sex steroids in lung health and disease. Endocr Rev.

[17] Cardenas L.Z., Santana P.V., Caruso P., Ribeiro de Carvalho C.R., Pereira de Albuquerque A.L. (2018). Diaphragmatic ultrasound correlates with inspiratory muscle strength and pulmonary function in healthy subjects. Ultrasound Med Biol.

[18] Sheel A.W., Dominelli P.B., Molgat-Seon Y. (2016). Revisiting dysanapsis: sex-based differences in airways and the mechanics of breathing during exercise. Exp Physiol.

[19] Dominelli P.B., Molgat-Seon Y. (2022). Sex, gender and the pulmonary physiology of exercise. Eur Respir Rev.

[20] Beduneau G., Pham T., Schortgen F., Piquilloud L., Zogheib E., Jonas M., Group WS, the RNdd (2017). Epidemiology of weaning outcome according to a new definition. The WIND study. Am J Respir Crit Care Med.

[21] Khazaei O., Laffey C.M., Sheerin R., McNicholas B.A., Pham T., Heunks L. (2025). Impact of comorbidities on management and outcomes of patients weaning from invasive mechanical ventilation: insights from the WEAN SAFE study. Crit Care.

[22] Lat T.I., McGraw M.K., White H.D. (2021). Gender differences in critical illness and critical care research. Clin Chest Med.

[23] Modra L.J., Higgins A.M., Pilcher D.V., Bailey M., Bellomo R. (2024). Sex differences in vital organ support provided to ICU patients. Crit Care Med.

[24] Modra L.J., Higgins A.M., Pilcher D.V., Bailey M.J., Bellomo R. (2022). Sex differences in mortality of ICU patients according to diagnosis-related sex balance. Am J Respir Crit Care Med.

[25] Roser E., Michels-Zetsche J.D., Ersoz H., Neetz B., Hoger P., Trinkmann F. (2024). Differences between women and men in prolonged weaning. Respir Res.

[26] Huang C. (2022). Gender differences in prolonged mechanical ventilation patients - a retrospective observational study. Int J Gen Med.

[27] Al-Bassam W., Parikh T., Neto A.S., Idrees Y., Kubicki M.A., Hodgson C.L. (2021). Pressure support ventilation in intensive care patients receiving prolonged invasive ventilation. Crit Care Resusc.

[28] Al-Bassam W., Dade F., Bailey M., Eastwood G., Osawa E., Eyeington C. (2019). “Likely overassistance” during invasive pressure support ventilation in patients in the intensive care unit: a multicentre prospective observational study. Crit Care Resusc.

[29] Akoumianaki E., Vaporidi K., Georgopoulos D. (2019). The injurious effects of elevated or nonelevated respiratory rate during mechanical ventilation. Am J Respir Crit Care Med.

[30] Saavedra S.N., Barisich P.V.S., Maldonado J.B.P., Lumini R.B., Gomez-Gonzalez A., Gallardo A. (2022). Asynchronies during invasive mechanical ventilation: narrative review and update. Acute Crit Care.

[31] von Wedel D., Redaelli S., Jung B., Baedorf-Kassis E.N., Schaefer M.S., Lung Injury G. (2025). Higher mortality in female versus male critically ill patients at comparable thresholds of mechanical power: necessity of normalization to functional lung size. Intensive Care Med.

[32] Monteiro A.C., Bartek B., Tripathi S., Calfee C.S., Matthay M.A., Sinha P. (2025). Short stature is an independent risk factor for ventilatory inefficiency and higher mortality in ARDS. Am J Respir Crit Care Med.

[33] Madotto F., Pham T., Bellani G., Bos L.D., Simonis F.D., Fan E. (2018). Investigators LS, the ETG. Resolved versus confirmed ARDS after 24 h: insights from the LUNG SAFE study. Intensive Care Med.

[34] Sasko B., Thiem U., Christ M., Trappe H.J., Ritter O., Pagonas N. (2018). Size matters: an observational study investigating estimated height as a reference size for calculating tidal volumes if low tidal volume ventilation is required. PLoS One.

